# The precuneus and posterior cingulate gyrus support temporal orientation in Alzheimer’s disease

**DOI:** 10.1093/braincomms/fcaf424

**Published:** 2025-10-29

**Authors:** Akinori Futamura, Ryuta Kinno, Yuki Hanazuka, Ryuta Ochi, Akira Midorikawa, Shigeru Kitazawa, Kenjiro Ono, Mitsuru Kawamura

**Affiliations:** Division of Neurology, Department of Medicine, Showa Medical University, Shinagawa-ku, Tokyo 142-8666, Japan; Department of Neurology, Showa Medical University Fujigaoka Hospital, Yokohama, Kanagawa 227-8501, Japan; Department of Neurology, Showa Medical University Fujigaoka Hospital, Yokohama, Kanagawa 227-8501, Japan; Department of Management Information, Hokkai Gakuen University, Sapporo, Hokkaido 062-8605, Japan; Department of Psychology, Graduate School of Letters, Chuo University, Hachioji, Tokyo 192-0393, Japan; Department of Psychology, Faculty of Letters, Chuo University, Hachioji, Tokyo 192-0393, Japan; Dynamic Brain Network Laboratory, Graduate School of Frontier Biosciences, The University of Osaka, Suita, Osaka 565-0871, Japan; Center for Information and Neural Networks (CiNet), National Institute of Information and Communications Technology, Suita, Osaka 565-0871, Japan; Division of Neurology, Department of Medicine, Showa Medical University, Shinagawa-ku, Tokyo 142-8666, Japan; Department of Neurology, Kanazawa University Graduate School of Medical Sciences, Kanazawa, Ishikawa 920-8640, Japan; Division of Neurology, Department of Medicine, Showa Medical University, Shinagawa-ku, Tokyo 142-8666, Japan

**Keywords:** Alzheimer’s disease, dementia, time perception, precuneus, posterior cingulate gyrus

## Abstract

Although the classifications of ‘past,’ ‘present,’ and ‘future’ are considered abstract concepts, we naturally understand them. Those classifications were named ‘A-series’ time by McTaggart in 1908. Alzheimer's disease (AD) is the most common type of dementia, with initial symptoms generally including temporal disorientation. This study aims to (1) elucidate the impairment process of temporal cognition in AD by administering A-series temporal tasks to individuals with AD, mild cognitive impairment (MCI), and healthy controls, and (2) clarify the relationship between temporal cognition at each stage of impairment and cerebral blood flow (CBF). A diagnosis of AD (n = 37), MCI (n = 10), and no dementia (ND) (n = 10) took part. The ‘A-series’ task consisted of eleven short sentences that were grammatically correct using seven-time qualifiers (last week, yesterday, today, now, tomorrow, this week, or next week). The participants were required to respond when the events in the sentences happened or would happen in nine stages. We compared the pattern of their responses, the scores of the Japanese version of the Mini-Mental State Examination (MMSE-J), and the regional CBFs performed by ^99m^Tc-ethyl cysteinate dimer Single Photon Emission Computed Tomography. We found that ND was intact in the ability to distinguish between the past, present, and future, on the other hand, AD and MCI showed a diminished ability in temporal orientation when we sorted the 11 sentences in the ascending order of the mean response scores among the ND participants, they were generally ordered according to the time represented by adverbs of time. We also found that the participants could be best classified into three clusters. All ND participants (10/10) and half of the MCI participants (5/10) belonged to Cluster 1, whereas only 19% of the AD participants belonged to the cluster (7/37). Cluster 2 was contributed by three MCI participants (3/10) and 30% of the AD participants (11/37). Finally, most of the AD participants (51%) belonged to cluster 3 (19/37) with a few MCI participants (2/10). We compared CBFs across the three clusters and found the CBF in the pairs of the left and the right pericallosal region could predict whether a participant belonged to either cluster at the largest hit rate of 75%. Our findings suggest that the bilateral pericallosal region, including the posterior cingulate gyrus and precuneus cortex, is associated with temporal orientation.

## Teaser

Futamura et al. report that the progression of temporal cognitive dysfunction in Alzheimer’s disease is associated with cerebral blood flow in the bilateral pericallosal regions, as measured by responses to verbal tense and temporal adverb stimuli.

## Graphical Abstract

**Graphical Abstract:**
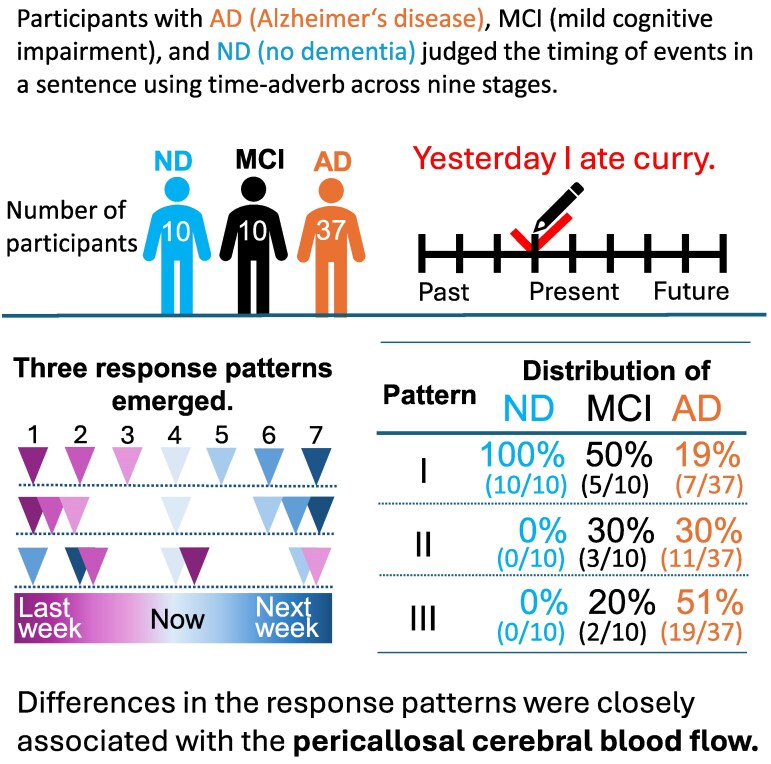

## Introduction

Although the classifications of ‘past,’ ‘present,’ and ‘future’ are considered abstract concepts, we naturally understand them.^1^ This continuous flow of time from past to future is referred to as ‘A-series time’.^2^ In a study involving young healthy individuals, Peer *et al*.^3^ presented two events and asked which was closer to the ‘present.’ They identified that temporal orientation is associated with brain regions including the bilateral precuneus and left parietal and temporal regions.

Alzheimer's disease (AD) is the most common type of dementia, with initial symptoms generally including temporal disorientation and episodic memory impairment.^4^ It is well-known that AD individuals are more prone than healthy older adults to make errors in identifying the current date, day of the week, and time.^5^ They are also more likely to exhibit confabulation or false memories when recalling past events and struggle with imagining future events.^6,7^

Due to these characteristics, questions about temporal orientation are widely used in brief assessments and staging classifications for AD. For example, the Mini-Mental State Examination (MMSE) and the revised Hasegawa's Dementia Scale (HDS-R) include questions on temporal orientation.^8,9^ Additionally, the Clinical Dementia Rating (CDR), used to classify the stages of AD, contains items assessing temporal disorientation.^10^

Recent research has shown that specific brain regions are involved in this temporal cognition. In AD, the posterior cingulate cortex has been associated with answering ‘current’ date, year, and day of the week.^11,12^ Patients with brain lesions in the precuneus showed similar dysfunctions on time-related questions.^13^ Furthermore, in AD when recalling episodic memories from the ‘past’ or imagining ‘future’ events, the posterior cingulate cortex is thought to be involved.^7^

However, it may be inappropriate to demand current time when evaluating temporal orientation in AD patients, due to their memory impairments. Tang *et al*.^14^ studied Japanese, Chinese, and English speakers, examining temporal cognition through sentences with temporal modifiers such as ‘yesterday,’ ‘today,’ and ‘tomorrow.’ Regardless of language, the ‘present’ tense consistently activated the precuneus regions.

This study aims to (1) elucidate the impairment process of temporal cognition in AD by administering A-series temporal tasks to individuals with AD, mild cognitive impairment (MCI), and healthy controls, and (2) clarify the relationship between temporal cognition at each stage of impairment and cerebral blood flow. This will provide further insights into the deterioration of temporal cognition in AD and MCI and its related brain regions, offering a better understanding of the neural mechanisms of temporal cognition.

## Materials and methods

### Participants

This study was approved by the Ethics Committees of Showa Medical University Hospital (clinical trial identifier number: 2164) and conducted according to the principles of the Declaration of Helsinki. All participants were registered in the memory clinic of Showa Medical University Hospital between 2017 and 2019. We administered the Japanese version clinical demented rating (CDR-J) and the Mini-Mental State Examination (MMSE-J).^8,10,15^

All met the core clinical criteria for probable AD dementia or mild cognitive impairment (MCI).^4,16^ Those who CDR-J was evaluated as above 1.0 were classified as AD, equal 0.5 was classified as MCI, and equal zero was as ND. Three ND and all MCI and AD participants underwent Single Photon Emission Computed Tomography (SPECT) examinations. Among them 18 participants (4 MCI and 14 Ad) underwent IMP-SPECT, and 32 participants (3 ND, 6 MCI and 23AD) underwent ECD-SPECT. Participants who underwent ECD-SPECT were selected for analysis, as this tracer was used more frequently and is the only one compatible with 3DSRT analysis.

### The A-series task

We prepared eleven short sentences in Japanese (supplementary data), which consisted of three parts: an adverb of time, a noun with a particle, and a verb. Let us take the sentence No.6: ‘昨日 カレーを 食べた =I ate a curry yesterday,’ for example. It starts with an adverb that points to the past (昨日= ‘yesterday’), which is followed by a noun with a particle (カレーを= ‘a curry’) and a final verb in the past tense (食べた = ‘ate’). We chose an adverb of time from seven: two related to the past (先週、昨日=　last week, yesterday), three related to the present (今、今日、今週 = now, today, this week), and two related to the future (明日、来週 = tomorrow, next week).

All eleven sentences were grammatically correct in Japanese. In Japanese, future events are typically expressed using the present tense. Thus, three sentences containing an adverb of the future were associated with a verb in the present form (No. 1, 4, 8). Four sentences with an adverb of the past were associated with a verb in the past tense (No. 3, 6, 7, 10). The remaining four sentences with an adverb of the present were associated with either a present-tense verb (No. 2, 9) or a past-tense verb (No. 5, 11). Participants were required to evaluate the time to which each sentence referred. They rated their evaluation by using a nine-point scale: they marked a 5 when a sentence felt like it had referred to the ‘present’, a 1 when it felt like it had referred to the ‘far past’, and a 9 when it felt like it had referred to the far future. In our previous study,^14^ by using 144 such sentences and the rating method, we showed that ratings were fairly stable across 54 participants of three different mother tongues though the ‘correct’ responses were not uniquely determined. It was noteworthy that sentences with an adverb of ‘Now’ was rated as 5 (present) with a probability greater than 0.9.^14^ Further, ratings of the normal participants reflected the relative order of the adverbs (such as ‘next week’ comes after ‘tomorrow’) and combinations of the adverbs and the tense of verbs.

### Cerebral blood flow analysis by 3DSRT analysis

#### Dynamic acquisition (discovery NM630; GE healthcare, Tokyo, Japan)

Participants lay in the supine position. Immediately after a bolus intravenous injection of 600 MBq of technetium-99 m ECD, dynamic acquisition was initiated using a gamma camera (Discovery NM630). A total of 120 frames were acquired at 1 s per frame using a 128 × 128 matrix (pixel size: 4.2 mm). The energy peak was set at 140 keV with an energy window of ±10%.

#### SPECT acquisition (GCA-9300R; canon medical systems, Tokyo, Japan)

Global cerebral blood flow (CBF) was non-invasively quantified using a Patlak plot,^17^ which is based on a two-compartment model with the following linear equation:


$$\frac{C_{T}(t)}{C_{P}(t)}=K_{1}\cdot \int_{0}^{t}\frac{C_{P}(\tau )}{C_{P}(t)}d\tau +V_{0}$$

Where:

-
$C_{T}(t)$ is the Tissue radioactivity concentration at time *t*.
-
$C_{P}(t)$ is the plasma radioactivity concentration at time *t*.
-
$K_{1}$ is the influx rate constant, corresponding to regional cerebral blood flow (rCBF).
-
$V_{0}$ is the initial distribution volume (the y-intercept of the linear plot).

By plotting $\frac{C_{T}(t)}{C_{P}(t)}$ against the normalized integral $\int_{0}^{t}\;\frac{C_{P}(\tau )}{C_{P}(t)}d\tau$, the slope of the linear portion of the plot yields the value of $K_{1}$, which is proportional to CBF.

Five minutes after the completion of the dynamic acquisition, SPECT imaging was performed using fan-beam collimation. Data were acquired using a continuous rotation method over 120°, with a 128 × 128 matrix (pixel size: 1.72 mm). The acquisition lasted 15 min using three detectors (30 views per detector, 6 repetitions, 150 s per cycle).

Scatter correction was performed using the triple energy window (TEW) method: the main energy window was centred at 140 keV with a width of ±10%, and a sub-window on the lower side was set at 7%. Image reconstruction was performed using the filtered back projection (FBP) method with a Butterworth pre-filter (order 8, cutoff frequency 0.50 cycles/cm). Attenuation correction was applied using the Chang method, with an attenuation coefficient of 0.15 cm⁻¹ and a threshold of 14% for contour extraction.

### 3DSRT Analysis

Regional cerebral blood flow (rCBF) in each ROI was calculated with the Three-Dimensional Stereotactic ROI Template (3DSRT), an automated ROI-based tool for brain perfusion SPECT.^18,19^

The 3DSRT processing pipeline includes the following steps:

1. Anatomic standardization of patient SPECT images to the Talairach stereotactic space using the SPM99 algorithm. A variant converted to the MNI space (termed ‘3DSRT-t-MNI’) is also available.
2. Automatic placement of 318 constant ROIs in each cerebral and cerebellar hemisphere (total 636 ROIs).
3. Calculation of rCBF in each ROI after global count normalization.
4. The 636 ROIs are grouped into 12 arterial territories: callosomarginal, precentral, central, parietal, angular, temporal, posterior (occipital), pericallosal, lenticular nucleus, thalamus, hippocampus, and cerebellum. Segmental CBF is obtained as the area-weighted mean of constituent ROIs.

Not that the original 3DSRT is based on the Talairach coordinate system; the MNI-based version is referred to as ‘3DSRT-t-MNI’ in subsequent literature.

### Statistical analysis

**Figure 1:**
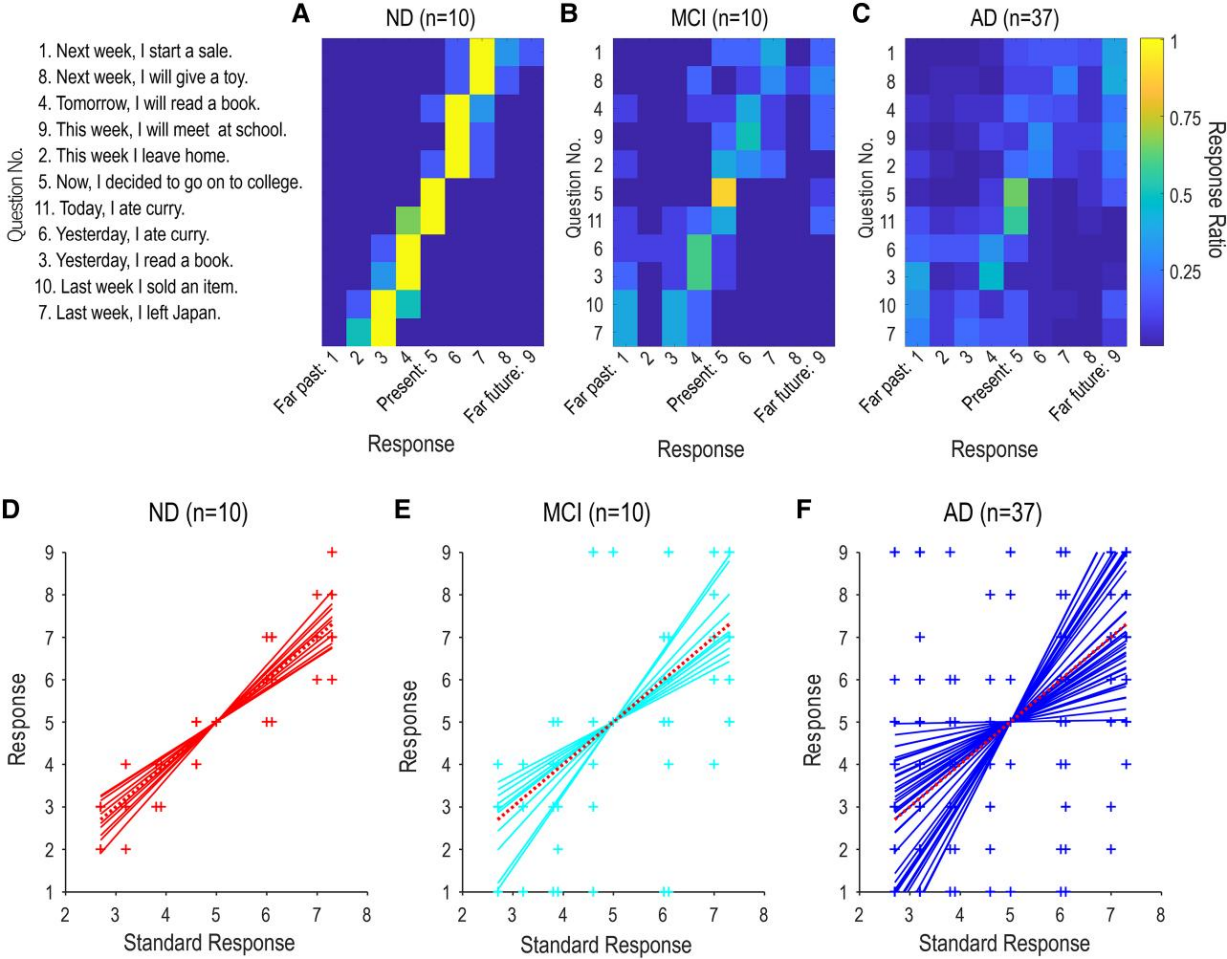
**Responses of the ND (A, D), MCI (B, E), and AD (C, F) participants. (A-C)** Each row of the tile plots shows the distribution of response scores to each one of the 11 sentences in the questionnaire. The 11 sentences are sorted in the ascending order of the mean response scores among the ND participants (standard response). Note that the score of ‘5’ represented the present, ‘1’ represented the far past, and ‘9’ represented the far future. Colours show the ratio of responses (0 to 1). **(D-F)** Each response to a sentence is plotted for each participant against the standard response of the sentence (crosses). Note that crosses overlap across participants due to the discreteness of responses (1 to 9). Regression lines calculated for each participant, constrained to pass through the pivotal present response point of (5, 5), are superimposed. A dotted red line represents the y = x.

Demographic comparisons across the study groups were analysed using one-way ANOVA followed by Tukey’s test for multiple comparisons with SPSS (ver. 27) software. We defined the standard response score of each sentence as the mean response score of the ND participants because the scores were generally stable across the ND participants (Fig. 1A). There were five ‘future’ sentences (No. 1, 8, 4, 9, 2), one ‘present’ sentence (No. 5), and five ‘past’ sentences (No. 11, 6, 3, 10, and 7). It is worth noting that all ND participants scored sentence No. 5 as being present (score 5). This shows that the sentence can be regarded as a pivotal sentence that represented the origin of time perception as reported previously.^14^

Eleven responses from each participant were then plotted against the standard response scores (Figs 1D-F). To quantify any deviation from the standard responses that passed the pivotal point of the present (5, 5) with a slope of one, we defined a line that passed through the pivotal point with a slope of k:


(1) y=k(x−5)+5.

The line was fitted to the responses of each participant and the estimated slope, and the determination coefficient (d.c., the ratio of the variance explained by the line) were recorded for further analyses. The standard response yields a slope of 1 and the determination coefficient of one.

**Figure 2:**
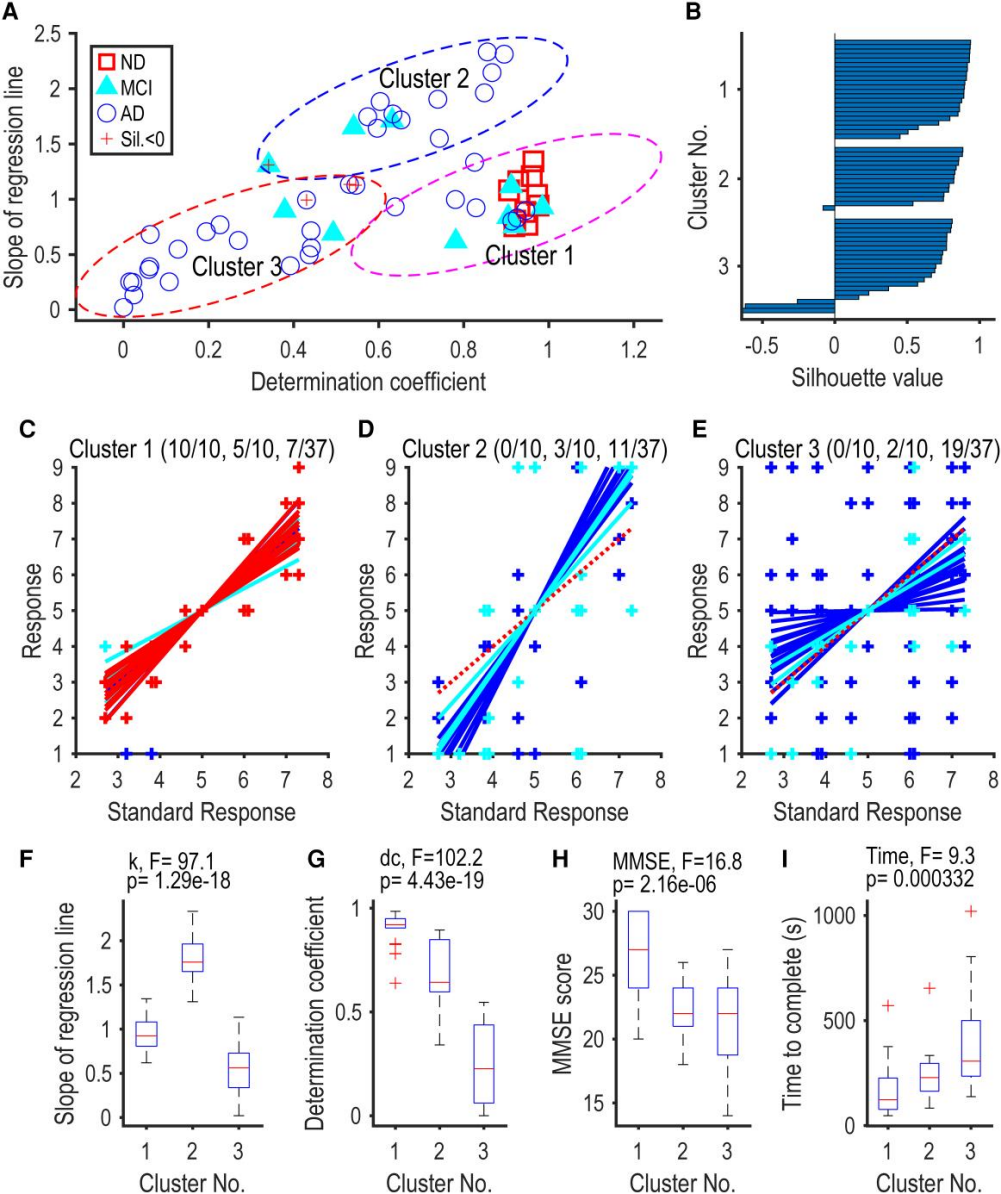
**Three clusters of the response patterns. (A)** Scatter plot of the slope of the regression line against the coefficient of determination (R²). Each symbol represents an individual participant: ND (red squares), MCI (cyan triangles), and AD (blue circles). Cluster analysis using a Gaussian mixture model classified the patterns into three distinct clusters, outlined by 95% confidence ellipses: Cluster 1 (magenta dashed ellipse), Cluster 2 (blue dashed ellipse), and Cluster 3 (red dashed ellipse). Note the minimal overlap among the ellipses. **(B)** Silhouette values for members of the three clusters, indicating the adequacy of clustering (values close to 1 denote good separation, negative values denote potential misclassification). **(C–E)** Response plots of participants belonging to Cluster 1 (**C**), Cluster 2 (**D**), and Cluster 3 (**E**). Regression lines are superimposed on individual data. Participant groups are colour-coded as ND (red), MCI (cyan), and AD (blue). Numbers in parentheses indicate the counts of ND, MCI, and AD participants in each cluster. **(F–I)** Across-cluster comparisons of (**F**) slope of the regression line, (**G**) coefficient of determination (R²), (**H**) Mini-Mental State Examination (MMSE) score, and (**I**) response time. Each boxplot represents data from 22 participants (Cluster 1), 14 participants (Cluster 2), and 21 participants (Cluster 3). F-values (F(2, 54)) and *P*-values from one-way ANOVA are indicated above each panel.

We then classified all participants into several clusters by fitting Gaussian mixture models to the two-dimensional data (slope versus d.c., Figure 2A). We used the mean Silhouette value, which takes the maximum value of one when the classification is perfect, to objectively determine the optimal number of clusters and to check the stability of allocation for each participant. Further, we drew an ellipse on the two-dimensional plane which showed the 95% confidence interval for each cluster (Mahalanobis distance = 2.45). All data analyses were performed by using functions implemented in MATLAB (R2021b, Mathworks).

## Results

### Demographic comparisons across the groups

The demographic data for ND, MCI, AD were analysed using one-way ANOVA followed by Tukey’s test for multiple comparisons (Table 1). There was no difference in age between the groups (F (2, 54) = 1.8, *P* = 0.17). The mean points of CDR-J increased on the order of ND, MCI, and AD. Their points were significantly different between ND versus AD, and MCI versus AD (F (2, 54) = 62.5, *P* < 0.01; ND versus MCI, *P* = 0.21; ND versus AD, *P* < 0.01; MCI versus AD, *P* < 0.01). The points of MMSE-J decreased on the order of ND, MCI, and AD. Their points were significantly different between the groups (F (2, 54) = 62.3, *P* < 0.01; ND versus MCI, *P* < 0.01; ND versus AD, *P* < 0.01; MCI versus AD, *P* < 0.01).

**Table 1:** Summary of background characteristics for each group

	Participants			*P* values
	ND	MCI	AD	
**Number of participants**	10	10	37	-
**Male: Female**	4: 6	5: 5	18: 19	-
**Age (mean ± SD)**	77.9 (± 5.0)	78.8 (± 5.8)	80.8 (± 4.3)	F (2, 54) = 1.8, *P* = 0.17
**CDR-J (mean ± SD)**	0.0 (± 0.0)	0.5 (± 0.0)	2.2 (± 0.8)	F (2, 54) = 62.5, *P* < 0.01 ND versus MCI, *P* = 0.21 ND versus AD, *P* < 0.01 MCI versus AD, *P* < 0.01
**MMSE-J (mean ± SD)**	29.9 (± 0.3)	25.7 (± 1.2)	20.9 (± 2.8)	F (2, 54) = 62.3, *P* < 0.01 ND versus MCI, *P* < 0.01 ND versus AD, *P* < 0.01 MCI versus AD, *P* < 0.01

The demographic data for ND (Not dementia), MCI (Mild cognitive impairment), AD (Alzheimer’s disease) were analysed using one-way ANOVA followed by Tukey’s test for multiple comparisons. There was no difference in age between the groups (F (2, 54) = 1.8, *P* = 0.17). The mean points of CDR-J increased on the order of ND, MCI, and AD. Their points were significantly different between ND versus AD, and MCI versus AD (F (2, 54) = 62.5, *P* < 0.01; ND versus MCI, *P* = 0.21; ND versus AD, *P* < 0.01; MCI versus AD, *P* < 0.01). The points of MMSE-J decreased on the order of ND, MCI, and AD. Their points were significantly different between the groups (F (2, 54) = 62.3, *P* < 0.01; ND versus MCI, *P* < 0.01; ND versus AD, *P* < 0.01; MCI versus AD, *P* < 0.01).

### The pattern analysis of the response of the A-series task

When we sorted the 11 sentences in the ascending order of the mean response scores among the ND participants, they were generally ordered according to the time represented by adverbs of time (the initial nouns) (Fig. 1A): last week (No.7, 2.5 ± 0.55; mean ± SD), last week (No.10, 3.2 ± 0.75), yesterday (No.3, 3.8 ± 0.41), yesterday (No.6, 3.8 ± 0.41), today (No.11, 4.5 ± 0.55), now (No.5, 5.0 ± 0), this week (No.2, 6.0 ± 0), this week (No.9, 6.0 ± 0), tomorrow (No.4, 6.2 ± 0.41), next week (No.8, 7.0 ± 0.63), next week (No.1, 7.2 ± 0.75). When adverbs of time were present (today, now, this week), the mean score was additionally determined by the tense of the verb. When ‘Today’ was followed by the past tense ‘ate’, the sentence (No. 11) was judged as the near past (score 4) or the present (score 5). When ‘now’ was followed by the past tense ‘decided’, the sentence (No. 8) was judged as the present (score 5) by all ND participants. This combination of the Japanese words corresponded to the present perfect in English. When ‘this week’ was followed by the present tense ‘leave’ or ‘meet’, the sentences were judged as belonging to the near future (score 6). The standard deviation of the response score increased to 0.75 at both ends where sentences started with ‘last week’ or ‘next week’, but responses of the ND participants were generally stable in that we could observe clear linear response patterns (Fig. 1A). The variability in the response increased in the MCI participants (Fig. 1B) but nine of them still judged the sentence No.5 as representing the present (score 5, 9/10). In AD participants, the response pattern became much more diverse although there remained some linear tendency (Fig. 1C).

To quantitatively characterize the response patterns of individual participants, we fitted a regression line to the data of each participant (Figs 1D-F). The ND participants yielded lines with slopes around one with small residual errors (Fig. 1D). The variability increased moderately in the MCI participants (Fig. 1E). In the AD participants, slopes of the regression line distributed widely from zero to 2.5 (Figure 1F).

To classify these variable response patterns, we further plotted the slope of the regression line against the determination coefficient (Fig. 2A). As expected, the ND participants (red square) formed a cluster around (1, 1) with a few MCI (cyan triangle) and AD (blue circle) participants. Interestingly, AD participants formed two clusters: one with the slope steeper than 1.5 and the determination coefficient greater than 0.5, and another with the slope smaller than 1 and the determination coefficient smaller than 0.6. These observations were supported by cluster analysis. By using the Gaussian mixture distribution model with the help of the Silhouette value, we found that the entire participants could be best classified into three clusters (Figs 2A and B). The classification was stable in that there was little overlap between the 95% confidence regions (ellipses in Fig. 2A). As a result, the Silhouette value was greater than 0.5 in the majority of the data (49/57 = 86%) except for a few data near the borders of the confidence regions.

The three clusters were characterized by nearly complete linear responses with slopes close to one (Cluster 1, Fig. 2C), fair linearity with slopes greater than 1.5 (Cluster 2, Fig. 2D), and poor linearity with slopes less than one (Cluster 3, Fig. 2E). It is worth noting that all ND participants (10/10) and the half of the MCI participants (5/10) belonged to Cluster 1, whereas only 19% of the AD participants belonged to Cluster 1 (7/37). Cluster 2 was contributed by three MCI participants (3/10) and 30% of the AD participants (11/37). Finally, the majority of the AD participants (51%) belonged to Cluster 3 (19/37) with a few MCI participants (2/10).

These observations suggest that the fine time participation, as observed in the Cluster 1 participants, was finally lost as AD progressed in the Cluster 3 participants, by way of a middle stage (Cluster 2) where the time was crudely scored in three grades of the far past (score 1), the present (5), and the far future (9). The MMSE-J scores dropped from the median of 27.0 in Cluster 1, to 22.4 and 20.6 in Clusters 2 and 3, (Fig. 2H; F (2, 54) = 21.3, *P* < 0.01; Cluster 1 versus Cluster 2, *P* < 0.01; Cluster 1 versus Cluster 3, *P* < 0.01; Cluster 2 versus Cluster 3, *P* < 0.01). Response time dropped from the median of 137.2 in cluster 1, to 274.7 and 391.9 in Clusters 2 and 3, (Fig. 2I; F (2, 54) = 12.6, *P* < 0.01; Cluster 1 versus Cluster 2, *P* < 0.05; Cluster 1 versus Cluster 3, *P* < 0.01; Cluster 2 versus Cluster 3, *P* = 0.09).

### The analysis of CBF across the clusters

**Figure 3:**
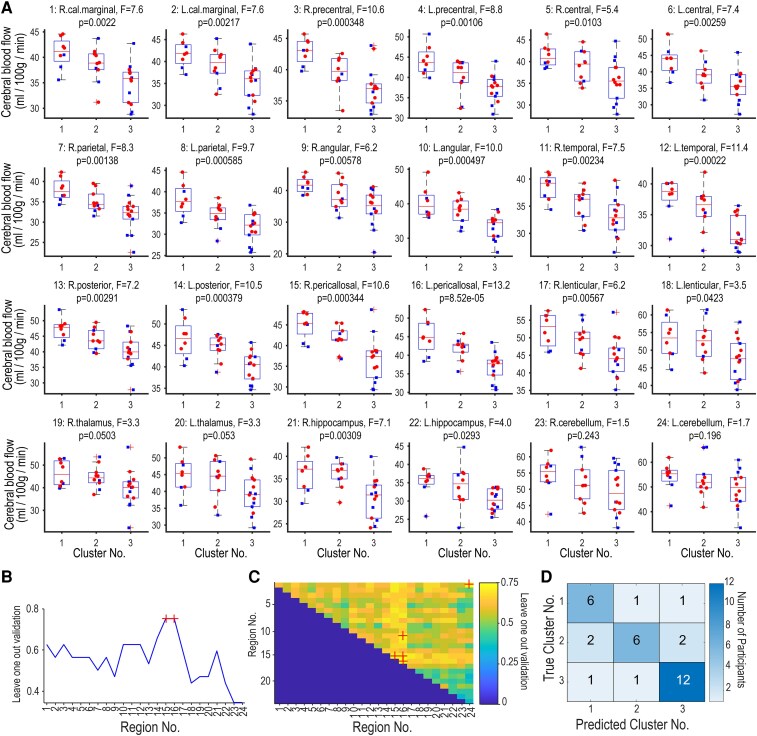
Comparison of regional cerebral blood flow (CBF) across Clusters 1–3. (**A**) Box plots show distributions of the CBF (ml/100 g/min) in 24 different brain regions in 3DSRT analysis (Takeuchi *et al*. 2003, 2006). *P* values of the one-way analysis of variance are shown at the top of each panel. Note that the CBF generally decreased in the order of Clusters 1, 2, and 3, each containing data from 8, 10, and 14 participants, respectively. Individual data points are shown by different symbols: blue squares for males and red circles for females. There were no significant sex differences in CBF in any of the 24 regions (Wilcoxon rank-sum test, FDR-corrected at 0.05). (**B**) The hit rate of the leave-one-out validation in the linear discriminant analysis. The analysis was repeated 24 times using the CBF of each of the 24 regions. The hit rate was maximized (0.75) when we used the CBF of the right (No. 15) and left (No. 16) pericallosal regions (red cross). (**C**) Hit rate of the leave-one-out validation in the linear discriminant analysis with two regional data. Each voxel represents a model with a pair of data. The colour shows the hit rate of the model (colour bar). Note that the best hit rate was again 0.75 and the pairs that yielded the best score (red cross) contained either No. 15 or No. 16. (**D**) A confusion matrix chart of one of the best models (single region model of No. 15). Rows show the true cluster number and columns show the predicted cluster number. The diagonal cells show the number of the hit (6, 6, and 12 for Clusters 1, 2, and 3). Abbreviations: R, right; L, left; cal. marginal, callosomarginal; lenticular, lenticular nucleus; No., number; 3DSRT, three-dimensional stereotactic ROI template. The region numbers in Figs 3B and C are consistent with those in Fig. 3A.

We finally compared CBFs across the three clusters in 24 brain regions (Fig. 3A). The CBF generally decreased in the order of Clusters 1, 2, and 3, each containing data from 8, 10, and 14 participants, respectively (Table 2). Regarding cardiovascular risk factors among the participants, 11 individuals had comorbid lifestyle-related diseases, including hypertension (n = 3), diabetes mellitus (n = 9), and dyslipidemia (n = 3), with some overlapping cases. In addition, 4 participants had a history of heart disease: atrial fibrillation treated with ablation (n = 1), angina pectoris (n = 2), and prior myocardial infarction (n = 1). All these individuals were receiving medical treatment at the time of the study.

**Table 2:** Summary of background characteristics for each cluster analysed by ECD-SPECT

	Cluster No.			*P* values
	1	2	3	
**Number of participants**	8	10	14	-
**Male: Female**	3: 5	3: 7	7: 7	-
**Age (mean** **±** **SD)**	82.8 (± 3.5)	77.0 (± 5.1)	82.3 (± 3.6)	F (2, 29) = 5.48, *P* = 0.01 Cluster 1 versus 2: *P* = 0.02 Cluster 1 versus 3: *P* = 0.97 Cluster 2 versus 3: *P* = 0.02
**CDR-J (mean** **±** **SD)**	0.7 (± 0.9)	1.7 (± 0.9)	2.4 (± 0.8)	F (2, 29) = 9.06, *P* < 0.01 Cluster 1 versus 2: *P* = 0.09 Cluster 1 versus 3: *P* < 0.01 Cluster 2 versus 3: *P* = 0.12
**MMSE-J (mean** **±** **SD)**	26.5 (± 3.4)	22.5 (± 2.3)	19.8 (± 3.5)	F (2, 29) = 10.4, *P* < 0.01 Cluster 1 versus 2: *P* = 0.04 Cluster 1 versus 3: *P* < 0.01 Cluster 2 versus 3: *P* = 0.14
**Response time (s, mean** **±** **SD)**	152.6 (± 75.8)	277.3 (± 137.6)	416.0 (± 177.7)	F (2, 29) = 7.83, *P* < 0.01 Cluster 1 versus 2: *P* = 0.22 Cluster 1 versus 3: *P* < 0.01 Cluster 2 versus 3: *P* = 0.09

The demographic data for each cluster were analysed using one-way ANOVA followed by Tukey’s test for multiple comparisons. The age was significantly different between Cluster 1 versus 2, and Cluster 2 versus 3 (F (2, 29) = 5.48, *P* = 0.01; Cluster 1 versus 2, *P* = 0.02; Cluster 1 versus 3, *P* = 0.97; Cluster 2 versus 3, *P* = 0.02). The mean points of CDR-J increased on the order of Cluster 1, 2, and 3. Their points were significantly different between Cluster 1 versus 3(F (2, 29) = 9.06, *P* < 0.01; Cluster 1 versus 2, *P* = 0.09, Cluster 1 versus 3, *P* < 0.01, Cluster 2 versus 3, *P* = 0.12). The mean points of MMSE-J decreased on the order of Cluster 1, 2, and 3. Their points were significantly different between Cluster 1 versus 2, and Cluster 1 versus 3 (F (2, 29) = 10.4, *P* < 0.01; Cluster 1 versus 2, *P* = 0.04; Cluster 1 versus 3, *P* < 0.01; Cluster 2 versus 3, *P* = 0.14). The mean response time increased on the order of Cluster 1, 2, and 3. Their times were significantly different between Cluster 1 versus 3 (F (2, 29) = 7.83, *P* < 0.01; Cluster 1 versus 2, *P* = 0.22, Cluster 1 versus 3, *P* < 0.01; Cluster 2 versus 3, *P* = 0.09).

**Figure 4:**
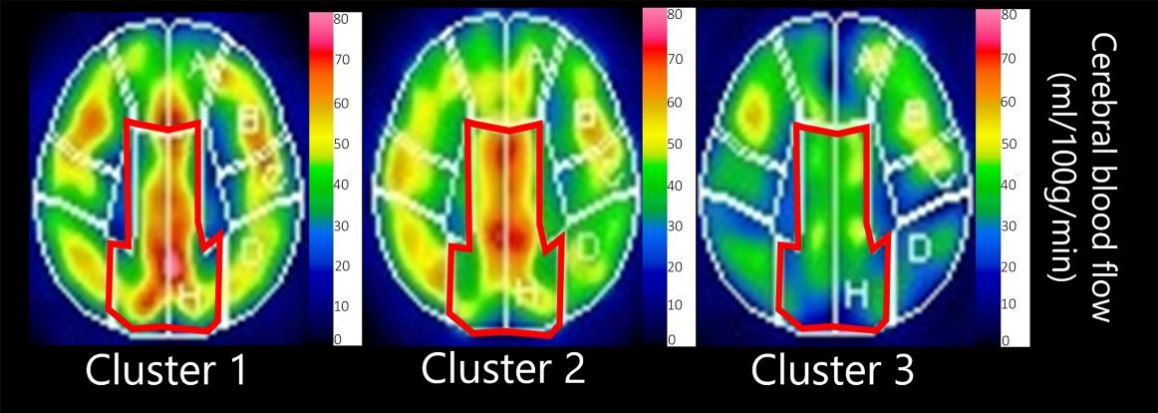
**Representative images of Pericallosal CBF.** CBF in the bilateral pericallosal region, labelled as region H in the 3DSRT analysis and outlined in red, decreased progressively across the clusters. The participant shown as an example of Cluster 1 was a 78-year-old man with AD, CDR-J 1, and MMSE-J 24. The example from Cluster 2 was a 77-year-old woman with AD, CDR-J 2, and MMSE-J 22. The example from Cluster 3 was a 75-year-old man with CDR-J 1 and MMSE-J 24. The pericallosal region includes the upper precuneus, posterior cingulate, and cingulate gyrus (Takeuchi *et al*., 2003, 2006).

In particular, nine of the 24 regions showed significant differences in the mean CBF across the three clusters after Bonferroni correction (one-way analysis of variance, *P* < 0.05/24). These regions were the left pericallosal, left temporal, right pericallosal, right precentral, left post-cerebral, left angular, left parietal, left precentral, and right parietal regions. We directly assessed whether the CBF in one single region could predict whether a participant belonged to either cluster by using the linear discriminant analysis and leave-one-out verification (Fig. 3B). We achieved the largest hit rate of 75% when we used the CBF in the left pericallosal or the right pericallosal region (Fig. 3B, red cross). We then expanded the model by using a pair of CBFs. We evaluated all 276 (= 24·23/2) pairs and found that no pair exceeded the best hit rate of 75% in the single region model, (75% of the Cluster 1, 60% of Cluster 2, and 85.7% of the Cluster 3) (Figs 3C and D). The representative images of the bilateral pericallosal CBF in each cluster were shown in Fig. 4. The pericallosal region includes the upper precuneus, posterior cingulate, and cingulate gyrus.^18,19^ We suggest that the bilateral pericallosal regions are important to achieve the fine perception of time across the past, the present, and the future.

## Discussion

Previous studies showed that temporal orientation is associated with specific brain regions. In healthy individuals, the left and right precuneus, the left inferior parietal lobule, which includes the angular gyrus, and the temporal regions, which include the superior temporal sulcus, have been implicated.^3,14^ In AD, the posterior cingulate cortex and precuneus have been identified.^7,11,12^ Our CBF analysis and these findings suggest that reduced CBF in the pericallosal regions which include the precuneus and posterior cingulate cortex is crucial for temporal orientation.

In AD and MCI, a decline in temporal cognition makes it difficult to recall past events, accurately perceive the flow of current time, and predict or plan for the future.^7^ Previously, we reported that AD patients perceive the flow of time subjectively, expressing feelings such as ‘living according to a private clock,’ ‘the past comes up, ‘‘moving back and forth between the present and the past, ‘cannot imagine the future,’ and ‘may bid farewell to this world as early as tomorrow’.^20^ According to the report by Skye *et al*.,^13^ two patients with a left precuneus lesion felt that ‘time did not run,’ and one of the two experienced a subjective time dilation, another had difficulty conceptualizing time. We also reported a case of left ACA region haemorrhagic infarction, where the patient felt that ‘time passing too quickly ‘and developed a compulsive routine to compensate.^21^ These findings suggest that the precuneus and posterior cingulate regions play an essential role in temporal orientation, the concept of time, and the subjective experience of time passing.

The results of this study indicate that individuals with AD and MCI have a diminished ability in accurate temporal orientation. As you can see from Figs 1A and D, in ND, there is a clear linear response pattern to each tense, indicating that the A-series temporal cognition, the ability to distinguish between the past, present, and future, remains intact. On the other hand, as shown in Figs 1B and E, in MCI, although responses to the present tense remain stable, the linearity of responses to the past and future tenses breaks down, showing a tendency to categorize them roughly. Finally, as seen in Figs 1C and F, in AD, the linearity of responses to all tense collapses.

The classification by temporal cognition showed a correlation with the AD classification of the staging of CDR. 50% of the MCI and 19% of the AD are categorized into cluster 1. 30% of the AD was categorized into cluster 2, and 51% of the AD was categorized into the cluster 3. The score of the MMSE-J and time to Response time dropped off between the clusters (Figs 2H and I). CDR comprises six domains: memory, orientation, judgment and problem-solving, community affairs, home and hobbies, and personal care. Among these, ‘memory’ has the most significant impact on staging.^10^ This finding reveals that the impairment patterns of temporal cognition do not align with the stage classifications of CDR, suggesting diversity in the temporal cognitive impairments in AD.

AD patients also showed deficits in ‘mental time travel”—the ability to mentally project themselves into the past or future.^22^ The temporal cognitive impairments in AD may be linked to the deterioration of episodic memory, highlighting the hippocampus’s role in this process.^23^ Although we designed this study to minimize the influence of episodic memory impairment, the relationship between disorientation and memory impairment cannot be completely ruled out. Memory is primarily processed by Papez's circuit, and the posterior cingulate has the neural connection between both the precuneus and the hippocampus. Yamashita *et al*.^12^ emphasized the importance of changes in neural connections within various networks centred around the posterior cingulate cortex in AD.

The background for the task design in this study is based on findings by Arzy *et al*.,^24^ who reported that temporal distinctions between past, present, and future spatially represented in the mind, as well as studies by Peer *et al*.^3^ and Tang *et al*.^14^ investigating event representation in terms of distance. From this perspective, the impairments observed in this task might reflect deficits in spatiotemporal representation rather than simply episodic memory impairment. The limitations of this study include the small sample size and the lack of evaluation of spatial impairments. We plan to address these issues in future studies.

Understanding the deterioration of temporal cognition and implementing appropriate interventions can be expected to improve the quality of life for AD patients. Future research should focus on elucidating the specific mechanisms of these impairments and developing effective care methods.

## Supplementary Material

fcaf424_Supplementary_Data

## Data Availability

All data used in this study are available as supplementary information file. All code used in this study are available at https://showa.repo.nii.ac.jp/records/2000532. Additional supporting materials can be obtained from the corresponding author upon reasonable request.
