# Supplementary material for: The precuneus and posterior cingulate gyrus support temporal orientation in Alzheimer’s disease
Source: Brain Commun. 2025 Oct 29;7(6):fcaf424. doi: 10.1093/braincomms/fcaf424 (PMC12641088; doi:10.1093/braincomms/fcaf424)
Supplement: fcaf424_Supplementary_Data [file fcaf424_supplementary_data.zip › Supplementary Data.docx]

**Supplementary data**

The task in Japanese English translation

No.1. 来週 セールを 開始する (Next week, I start a sale.)

No.2 今週 実家を 出る (This week, I leave home.)

No.3 昨日 本を 読んだ (Yesterday, I read a book.)

No.4 明日 本を 読む (Tomorrow, I will read a book.)

No.5 今 進学を 決めた (Now, I decided to go on to college.)

No.6 昨日 カレーを 食べた (Yesterday, I ate curry.)

No.7 先週 日本を 出発した (Last week, I left Japan.)

No.8 来週 おもちゃを あげる (Next week, I will give a toy.)

No.9 今週 学校に 集まる (This week, I will meet at school.)

No. 10 先週 商品を 売った (Last week I sold an item.)

No. 11 今日 カレーを 食べた (Today, I ate curry.)
